# Knowledge, attitude and practice of healthy lifestyle among primary care registered population in Qatar: A cross sectional study

**DOI:** 10.1177/22799036261457516

**Published:** 2026-06-05

**Authors:** Mohamed Ahmed Syed, Ahmed Sameer Al Nuaimi, Abduljaleel Abdullatif Zainel, Hafiz Ahmed Mohamed, Muslim Abbas Syed

**Affiliations:** 1 Department of Clinical Research, 420951 Directorate of Clinical Affairs, Primary Health Care Corporation, Doha, Qatar

**Keywords:** healthy lifestyle, primary care, physical activity, diet, cross sectional study

## Abstract

**Background:**

Non-communicable diseases (NCDs) are a major contributor to morbidity and mortality worldwide, with Qatar experiencing a particularly high burden. Lifestyle-related risk factors, including physical inactivity and poor dietary habits, are central to NCD prevalence. Understanding the knowledge, attitudes, and practices (KAP) regarding healthy lifestyle behaviours is essential for designing effective public health interventions.

**Aim:**

To evaluate KAP related to two key components of healthy lifestyle—healthy diet and physical activity among adults using primary care services in Qatar.

**Methods:**

A cross-sectional survey was conducted among 940 adults registered with the Primary Health Care Corporation (PHCC). Participants were selected using multistage random sampling across PHCC. Data were collected using a culturally adapted, interview-based questionnaire assessing healthy diet and physical activity. KAP scores were standardised to a 0–100 scale.

**Results:**

Participants demonstrated high knowledge (healthy diet: mean 75.7; physical activity: mean 66.6) and positive attitudes (healthy diet: mean 74.1; physical activity: mean 76.9) towards healthy lifestyle behaviours. However, practice scores were substantially lower (healthy diet: mean 49.6; physical activity: mean 9.6). No significant associations were found between healthy diet practice and gender, BMI, or educational status, while age, nationality, marital status, and knowledge and attitude scores were significantly associated with healthy diet practice.

**Conclusion:**

Despite adequate knowledge and favourable attitudes, the adoption of healthy lifestyle practices remains limited among Qatar’s primary care population. These findings highlight the need for targeted interventions that address behavioural barriers and facilitate the translation of knowledge into practice. The insights gained are critical for informing public health policies and NCD prevention efforts in Qatar.

## Introduction

Non-communicable diseases (NCDs) are a leading cause of morbidity and mortality globally.^1^ According to the World Health Organization (WHO), 41 million people die annually (approximately 74% of all deaths) as a result of NCDs annually.^2^ Of these cardiovascular diseases account for the highest (17.9 million) followed by cancers (9.3 million), chronic respiratory diseases (4.1 million), and diabetes (2 million).^2^

Lifestyle related risk factors such as physical inactivity, poor dietary habits, smoking are significantly associated with NCDs.^1,3,4^ With the newer and more advanced pharmaceutical interventions becoming available, there is an increase in the number of individuals living with chronic conditions.^4^ This has resulted in increasing healthcare costs.^5^ Adopting a healthy lifestyle considered the most cost-effective approach to prevention of NCDs.^6^

As in most countries, NCDs are a leading cause of morbidity and mortality in Qatar. The country has early onset and high prevalence of NCDs. It has been reported that 16% of the population registered with publicly funded primary care settings have at least one NCD.^7^ NCDs contribute to 68% of all-cause mortality in Qatar.^8^ Annually, they cost approximately USD 2 billion in healthcare expenditure and USD 3 billion in lost productive capacities due to premature mortality, disability and workplace losses.^9^ These costs equate to approximately 2.7% of Qatar’s 2019 Gross Domestic Product (GDP).^9^

Trends show NCDs are on the rise in Qatar.^10,11^ They are likely to pose greater challenges in the future if not addressed urgently. Population level interventions to address NCDs are well established. The WHO has identified and recommends interventions that are feasible and cost-effective to prevent and manage NCDs.^10^ However, to effectively tailor and implement them, it is necessary to consider what is known and done about healthy lifestyle behaviours. KAP pertaining to healthy lifestyle plays an important role in prevention and management of NCDs. KAP elements are interdependent on each other. Therefore, a survey of knowledge, attitude, and practice (KAP) of healthy lifestyle behaviors was conducted in Qatar’s primary care registered population. The aim of the study was to evaluate KAP related to two key components of healthy lifestyle—healthy diet and physical activity among adults using primary care services in Qatar. . These findings will provide healthcare decision makers and policy makers with essential information when considering population level interventions for NCD prevention and control.

## Methodology

### Study design

**Figure 1.**
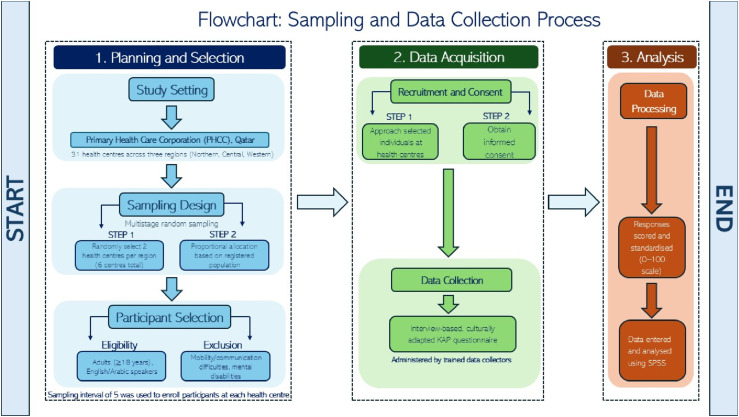
Study flowchart.

A cross-sectional study design was employed (See Figure 1).

### Study settings and participants

The study was conducted in Primary Health Care Corporation (PHCC), a publicly funded organization and the largest primary care provider in Qatar. PHCC has 31 primary health care centers located across three geographic regions (Northern, Central and Western) of the country, all of which are accredited by Accreditation Canada. Majority (70%) of the Qatar’s population is registered with PHCC.

Individuals aged 18 or above who could communicate in English or Arabic were eligible for inclusion in the study. Individuals with difficulties related to mobility and communication and mental disabilities were excluded.

A multistage systematic random sampling technique was adopted. Two health centres were randomly selected from each of the three geographic region. The total sample size was proportionately divided between the six health centres in accordance with the population registered in each of the three geographic regions.

The sample size was calculated based on the formula n = Z^2^_1-_α * p * (1 - p)/e^2^ [where Z = 1.96 for a confidence level (α) of 95%, p = proportion and e = margin of error]. Assuming p= 0.5, it a sample size of 365 was estimated. To allow for subgroup analysis, the sample size was multiplied by 3 to define a study sample of 1095. A systematic random sample with a sampling interval of 5 was used to enroll participants at each study location.

### Questionnaire and data collection

The study authors developed and tailored to understand KAP of healthy lifestyle within the context of Qatar primary care settings by reviewing existing surveys, adaptation to local context, expert consultation and feedback, pilot testing, feedback analysis, cognitive interviews and translation and validation. The authors have published the details of the development and validation of the questionnaire else where.^12^ The questionnaire included a total 69 close ended questions (multiple choice or 5- point Likert scale) across three sections – Sociodemographic characteristics, health diet and physical activity (Table 1).

**Table 1.** Number of sections and questions.

Section	Sub-section	Number of questions
Sociodemographic characteristics	Not applicable	14
Healthy diet	Knowledge	6
	Attitude	16
	Practice	13
Physical activity	Knowledge	3
	Attitude	7
	Practice	10
Total		69

Trained data collectors approached individuals visiting study locations and invited them to participate. If participants agreed, informed consent was obtained, and an interview-based questionnaire was administered in English and Arabic. The interview took approximately 70 minutes per participant to complete.

### Study locations

The study was conducted at two randomly selected PHCC health centres from each of the three geographic regions of Qatar as follows - Central Region (Al-Wakra & Airport health centres), Western Region (Mesaimeer & Al Rayyan health centres) and Northern region (Al Khor & Leabaib health centres).

### Data analysis

A total of 90 items were identified in the questionnaire (See Table 2). Items were given a score of one if favorably answered. A score was calculated by summing the total score for each element (knowledge, attitude and practice) in both healthy lifestyle categories (healthy diet and physical activity). The resulting scores were then rescaled and standardized to obtain a maximum possible score of 100 for each of the three elements in the two categories resulting in 6 scores in total.

**Table 2.** Number of responses per category.

Category	Element	Number of items	Total
Healthy diet	Knowledge	49	72
	Attitude	16	
	Practice	7	
Physical activity	Knowledge	7	18
	Attitude	7	
	Practice	4	
Total		**90**	

Quality assurance practices were employed to reduce missing data to a manageable amount. To retain the credibility of the calculated results missing data were not manipulated or imputed (Supplemental file Table S1). All data were analysed using the IBMSPSS ver 28 ‘Statistical Package for the Social Sciences (SPSS)’ statistical software package. The results were expressed as frequency and percentage distribution in the case of categorical variables, and as a range, mean, standard deviation (SD) and standard error (SE) for quantitative variables. Compliance of a continuous quantitative random variable with Gaussian curve (normal distribution) was analyzed using the Kolmogorov-Smirnov test. The statistical significance of difference in mean of a normally distributed continuous outcome response variable (score) between two groups was assessed by independent samples t-test, while between more than two groups ANOVA test was used. The quintile method was used to convert scores from a quantity to an ordinal variable with elements using the unbiased quartile approach. Multiple regression models were used to measure the adjusted association of a set of explanatory variables on a quantitative outcome variable (practice score). An estimate was considered statistically significant if its P value was less than an α level of significance of 0.05 in a two-sided test.

### Ethical considerations

The study presented a minimal risk of harm to its subjects, and the data collected for it were anonymized. The voluntary participation of recruited individuals was ensured by securing signed informed consent. None of the subjects’ personal information was collected nor was available to the research team. Overall, the study was conducted with integrity according to generally accepted ethical principles and was approved by the PHCC’s Institutional Review Board (Ref no. PHCC/DCR/2022/09/054).

## Results

### Study population

A total of 940 individuals participated in the study. Of these majority were aged 30-39 years old, 56.1% were women, 42.1% were of Southern Asian nationality and 74.5% were married. 66.5% of participants were either overweight or obese. 86.2% were educated to secondary school or higher level. In addition, five comorbidities were assessed. These included asthma, cardiovascular diseases (heart attack or angina or a stroke), raised blood pressure (hypertension), diabetes mellitus, and raised blood cholesterol (dyslipidemia). The high blood pressure was the most frequently reported (16.5%), while asthma was the least frequent (3.8%) (Table 3).

**Table 3.** Study population by age, gender, nationality, educational status, marital status and BMI.

Sociodemographic information	N	%
**Age (years)**		
<30	195	21.7
30-39	389	43.4
40-49	200	22.3
50-59	88	9.8
60+	25	2.8
**Gender**		
Women	513	56.1
Men	402	43.9
**Nationality**		
Qatari	234	24.9
South-eastern Asia	129	13.7
Southern Asia	401	42.7
Other	159	16.9
Unknown	17	1.8
**Marital status**		
Never married before	205	22.2
Married	688	74.5
Separated/Divorced/Widow	30	3.3
**Educational status**		
Did not complete the secondary school)	128	13.8
Completed secondary school/Trade/technical/vocational qualification	228	24.5
University Diploma/Bachelor degree/Postgraduate degree	573	61.7
**BMI (Kg/m2)**		
Underweight (<18.5)	13	1.4
Normal (18.5-24.9)	291	32.1
Overweight (25-29.9)	405	44.7
Obese Grade-I (30-34.9)	132	14.6
Obese Grade-II (35-39.9)	40	4.4
Obese Grade-III (40+)	25	2.8
**Comorbidities**		
Asthma	98	10.4
Cardiovascular Diseases (heart attack, angina, stroke)	36	3.8
Raised blood pressure (hypertension)	155	16.5
Diabetes Mellitus	105	11.2
Raised blood cholesterol (dyslipidemia)	122	13.0

N= number of participants.

### Healthy diet

**Figure 2.**
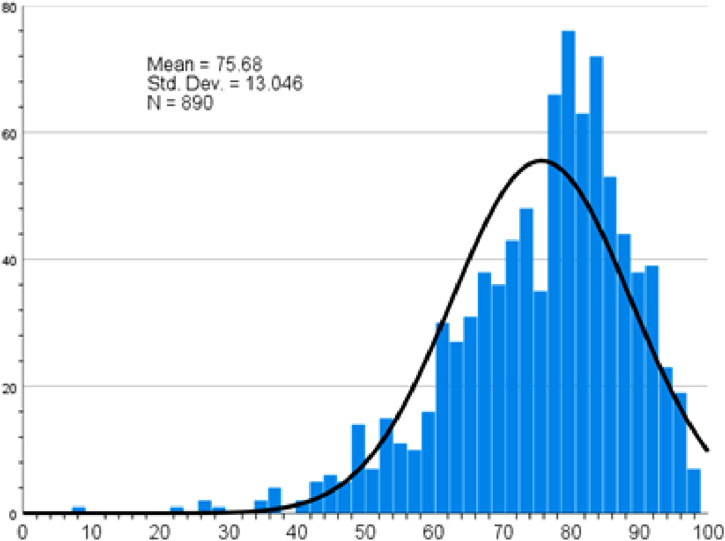
Healthy diet knowledge score.

Overall, participants demonstrated a mean knowledge score of 75.68 related to healthy diet (Figure 2). Participants demonstrated least knowledge regarding reading food labels to choose nutritious foods with high fiber content and to avoid bad fat (58.7%), reading food labels to avoid food with high calorie/energy content (54.9%), choosing sources other than dairies as a source of calcium and vitamin D rich foods like almonds and chickpeas (54.8%), avoiding carbohydrate rich food (51.2%); recognising osteoporosis (54.6%) and degenerative joint problems (53.3%) as a health risk associated with obesity and unhealthy food; and recognising foods such as laban, mortadella, salami and sausages as high in salt content (Supplemental file Table S2).

**Figure 3.**
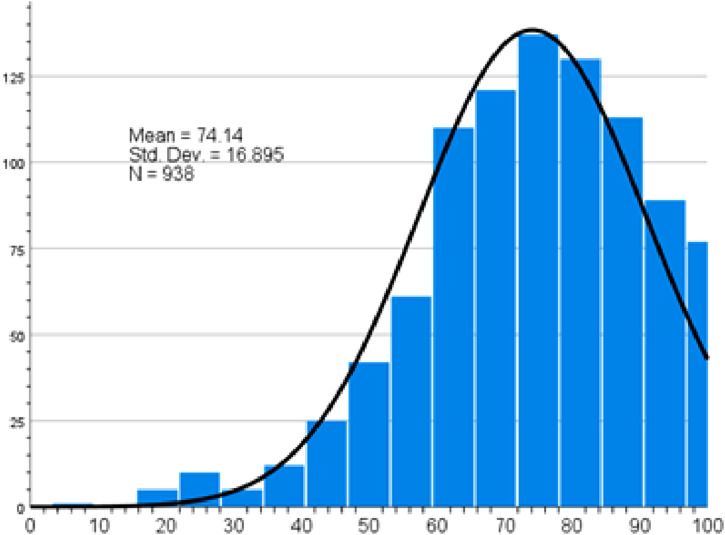
Healthy diet attitude score.

Participants in general demonstrated a mean attitude score of 76.9 towards healthy diet (Figure 3). Least positive attitude was towards healthy food not being tasty (51.4%), not letting children eat junk food (46.5%) and thinking about calories when eating (37.8%) (Supplemental file Table S3).

**Figure 4.**
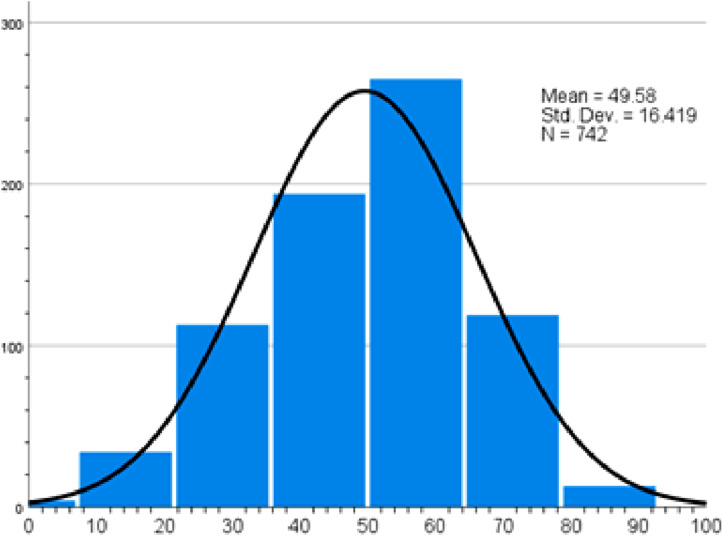
Healthy diet practice score.

A mean adherence score of 49.58 towards practicing healthy dietary habits (Figure 4) was seen amongst participants. Drinking natural fruit juice or vegetable juice less than once a week (32.5%), consuming recommended portions of vegetables (8.2%) and fruits (4.6%) were the least adhered practices (Supplemental file Table S4).

Gender, Body Mass Index (BMI) and educational status had no statistically significant association with the mean total score of healthy diet practice. Age, nationality, marital status, total diet knowledge score and total diet attitude score were significantly associated with mean total score of healthy diet practice. In addition, among comorbidities, only diabetes was associated with a significantly higher healthy diet practice score (Table 4).

**Table 4.** Association of healthy diet practice score with sociodemographic status, comorbidities, physical activity knowledge and attitude.

​	Total healthy diet practice score (max=100)					
	Range	Mean	SD	SE	N	P Value
**Age (years)**	​	​	​	​	​	<0.001
<30	(0 - 85.7)	44.4	17.5	1.42	153	​
30-39	(14.3 - 85.7)	50.1	15.8	0.90	307	​
40-49	(0 - 85.7)	52.7	14.1	1.12	158	​
50+	(14.3 - 85.7)	53.0	16.8	1.74	94	​
**Gender**	​	​	​	​	​	0.49[NS]
Female	(0 - 85.7)	49.9	16.8	0.83	408	​
Male	(0 - 85.7)	49.1	16.0	0.90	317	​
**Nationality**	​	​	​	​	​	0.001
Qatari	(0 - 85.7)	48.2	18.0	1.27	199	​
South-eastern Asia	(14.3 - 85.7)	45.4	16.6	1.65	102	​
Southern Asia	(14.3 - 85.7)	52.1	14.2	0.81	304	​
Other	(0 - 85.7)	49.0	17.8	1.52	137	​
**BMI (≥30 kg/m2)**	​	​	​	​	​	0.3[NS]
Non-obese (<30)	(0 - 85.7)	50.1	16.1	0.68	560	​
Obese (30+)	(0 - 85.7)	48.5	17.2	1.37	158	​
**Marital status**	​	​	​	​	​	<0.001
Never married	(0 - 85.7)	43.8	17.2	1.35	162	​
Ever married	(0 - 85.7)	51.3	15.8	0.66	566	​
**Educational status**	​	​	​	​	​	0.38[NS]
Less educated (did not complete the secondary school)	(0 - 71.4)	50.7	16.8	1.72	96	​
Completed 12 classes/Trade/technical/vocational qualification	(0 - 85.7)	48.3	16.0	1.17	189	​
University Diploma/Bachelor degree/Postgraduate degree	(0 - 85.7)	50.0	16.6	0.78	447	​
**Total diet knowledge score (max=100)**	​	​	​	​	​	0.035
Lowest (First) quartile (<= 67.3)	(0 - 71.4)	43.3	15.9	1.44	122	​
Average (interquartile range) 67.4 - 85.7	(0 - 85.7)	51.2	15.3	0.77	392	​
Highest (Fourth quartile) 85.8+	(0 - 85.7)	48.8	18.4	1.34	189	​
**Total physical activity attitude score (max=100)**	​	​	​	​	​	<0.001
Lowest (First) quartile (<= 62.5)	(0 - 71.4)	44.3	16.0	1.18	185	​
Average (interquartile range) 62.6 - 87.5	(0 - 85.7)	49.7	16.4	0.81	412	​
Highest (Fourth quartile) 87.6+	(14.3 - 85.7)	55.9	14.9	1.24	144	​
**Asthma**	​	​	​	​	​	0.21[NS]
Negative	(0 to 85.7)	49.8	16.2	0.62	682	​
Positive	(0 to 71.4)	46.7	18.8	2.42	60	​
**Cardiovascular Diseases (heart attack or angina or a stroke)**	​	​	​	​	​	0.82[NS]
Negative	(0 to 85.7)	49.5	16.2	0.61	720	​
Positive	(14.3 to 85.7)	50.6	21.9	4.67	22	​
**Raised blood pressure (hypertension)**	​	​	​	​	​	0.29[NS]
Negative	(0 to 85.7)	49.3	16.3	0.65	628	​
Positive	(14.3 to 85.7)	51.1	16.9	1.58	114	​
Diabetes	​	​	​	​	​	0.031
Negative	(0 to 85.7)	49.1	16.3	0.63	661	​
Positive	(14.3 to 85.7)	53.4	16.9	1.87	81	​
**Raised blood Cholesterol (dyslipidemia)**	​	​	​	​	​	0.09[NS]
Negative	(0 to 85.7)	49.2	16.2	0.64	650	​
Positive	(14.3 to 85.7)	52.5	17.7	1.85	92	​

SE= Standard Error, SD= Standard Deviation, N= number of participants, BMI= Body Mass Index, NS-= not statistically significant.

Among the list of explanatory variables tested for its prediction of the practice score in a multivariate regression model the attitude score had the strongest net association with a positive increase practice after adjusting for the remaining explanatory variables included in the model. Additionally, increasing age, and being married significantly increased the practice score. The model was statistically significant and able to explain 12.8% of the total variation in the dependent outcome variable (Supplemental file Table S5).

### Physical activity

**Figure 5.**
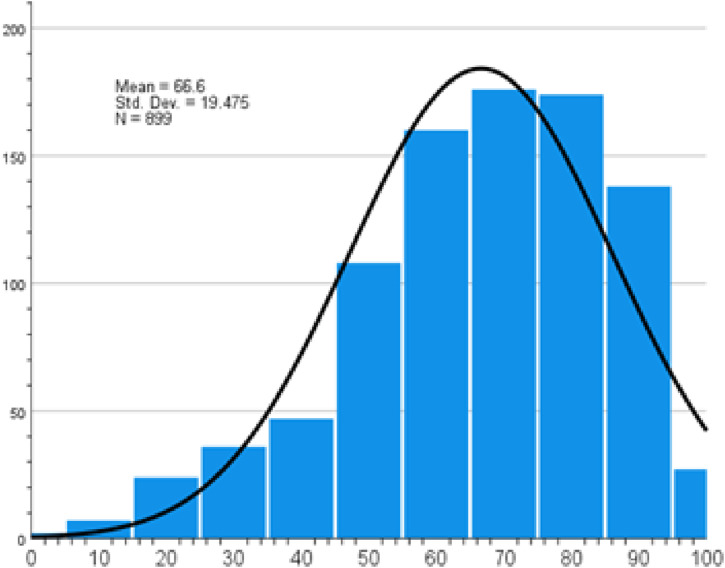
Physical activity knowledge score.

Overall, participants demonstrated a mean knowledge score of 66.6 about physical activity (Figure 5). The least knowledge levels were regarding identification of intensity aerobic physical activity (like running and swimming) for a minimum of 20 minutes a day for 3 days per week (37.4%) (Supplemental file Table S6).

**Figure 6.**
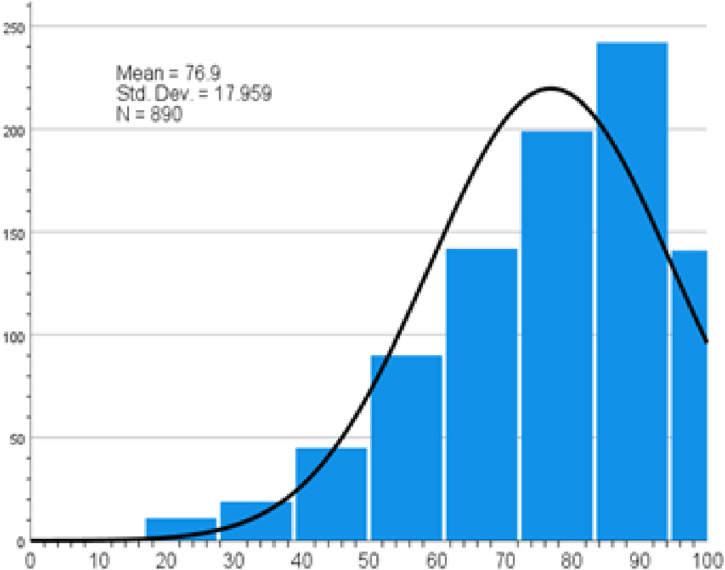
Physical activity attitude score.

Participants demonstrated a mean attitude score of 76.9 towards physical activity (Figure 6). The favorable attitude was towards restricting TV, electronic games, smart phone and computer use (Supplemental file Table S7).

**Figure 7.**
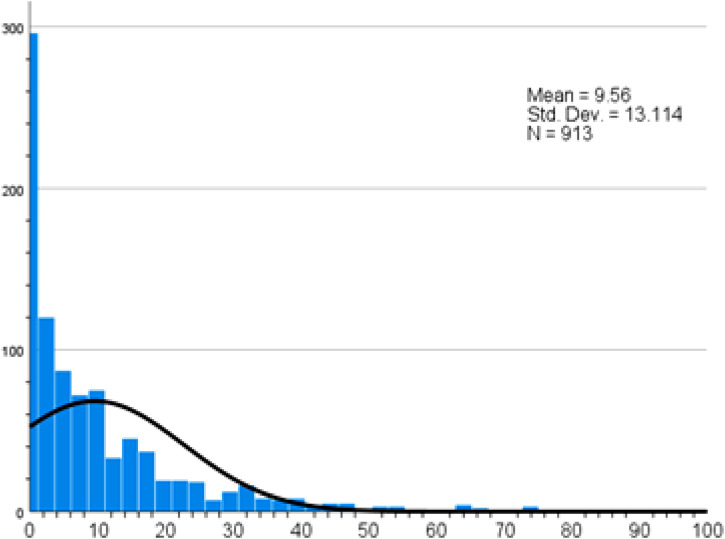
Physical activity practice score.

The mean score for practice of physical activity was 9.56 (Figure 7). 23.9% and 50.5% participants did not engage in any or low intensity physical activity respectively (Supplemental file Table S8).

Age, gender, BMI, marital status, and total knowledge of diet score had no statistically significant association with the mean total score of healthy diet practice. Age, nationality, total diet knowledge score and total diet attitude score were significantly associated with mean total score of healthy diet practice. In addition, none of the five comorbidities had any statistically significant association with healthy diet practice score (Table 5).

**Table 5.** Association of physical activity practice score with sociodemographic status, comorbidities, physical activity knowledge and attitude.

​	Total physical activity practice score (max=100)					
	Range	Mean	SD	SE	N	P Value
**Age (years)**						
<30	(0 - 100)	9.9	14.2	1.03	190	0.13[NS]
30-39	(0 - 73.9)	9.5	12.8	0.66	378	​
40-49	(0 - 73.9)	10.7	13.7	0.99	193	​
50+	(0 - 61.6)	7.0	11.4	1.09	110	​
**Gender**						
Female	(0 - 73.9)	9.8	13.1	0.59	501	0.29[NS]
Male	(0 - 100)	8.9	12.8	0.65	387	​
**Nationality**						
Qatar	(0 - 61.6)	7.7	10.3	0.68	229	0.007
South-eastern Asia	(0 - 73.9)	8.5	12.0	1.08	124	​
Southern Asia	(0 - 100)	9.8	14.0	0.71	390	​
Other	(0 - 66.5)	12.2	14.6	1.12	170	​
**BMI (≥30 kg/m2)**						
Non-obese (<30)	(0 - 100)	9.9	13.4	0.51	687	0.08[NS]
Obese (30+)	(0 - 65)	8.2	11.8	0.85	193	​
**Marital status**						
Never married	(0 - 100)	11.4	15.8	1.12	201	0.05[NS]
Ever married	(0 - 73.9)	9.1	12.3	0.46	696	​
**Educational status**						
Did not complete the secondary school	(0 - 57.6)	5.9	10.2	0.92	125	<0.001
Completed secondary school/Trade/technical/vocational qualification	(0 - 53.2)	8.4	10.8	0.73	219	​
University Diploma/Bachelor degree/Postgraduate degree	(0 - 100)	10.8	14.2	0.60	558	​
**Total physical activity knowledge score (max=100)**	​	​	​	​	​	<0.001
Lowest (First) quartile (<= 60.0)	(0 - 65)	7.9	11.0	0.57	372	​
Average (interquartile range) 61.0 - 80.0	(0 - 100)	10.3	14.6	0.78	345	​
Highest (Fourth quartile) 81.0+	(0 - 73.9)	12.6	14.3	1.13	161	​
**Total physical activity attitude score (max=100)**						
Lowest (First) quartile (<= 66.7)	(0 - 100)	7.4	12.5	0.73	296	<0.001
Average (interquartile range) 66.8 - 88.9	(0 - 73.9)	9.5	12.5	0.60	436	​
Highest (Fourth quartile) 89.0+	(0 - 73.9)	15.6	15.5	1.32	138	​
**Asthma**	​	​	​	​	​	0.08[NS]
Negative	(0 to 100)	9.3	12.8	0.45	818	​
Positive	(0 to 73.9)	12.1	15	1.54	95	​
**Cardiovascular Diseases (heart attack, angina, stroke)**	​	​	​	​	​	0.1[NS]
Negative	(0 to 100)	9.7	13.2	0.45	878	​
Positive	(0 to 40.9)	6.6	10.6	1.8	35	​
**Raised blood pressure (hypertension)**	​	​	​	​	​	0.86[NS]
Negative	(0 to 100)	9.6	13.2	0.48	761	​
Positive	(0 to 61.6)	9.4	12.9	1.05	152	​
Diabetes	​	​	​	​	​	0.54[NS]
Negative	(0 to 100)	9.6	13.3	0.47	809	​
Positive	(0 to 55.2)	8.9	11.3	1.11	104	​
**Raised blood Cholesterol (dyslipidemia)**	​	​	​	​	​	0.79[NS]
Negative	(0 to 100)	9.5	13.1	0.47	792	​
Positive	(0 to 55.2)	9.9	13	1.18	121	​

SE= Standard Error, SD= Standard Deviation, N= number of participants, BMI= Body Mass Index, NS-= not statistically significant.

Among the list of explanatory variables tested for its prediction of the practice score in a multivariate regression model, the attitude score had the strongest net association with a positive increase practice score after adjusting for the remaining explanatory variables included in the model. Additionally, being married significantly decreased the practice score, while a higher education significantly increased the outcome score. The model was statistically significant and able to explain 9.8% of the total variation in the dependent outcome variable (Supplemental file Table S9).

## Discussion

NCDs are on the rise in Qatar and globally. Healthy lifestyle is critical in preventing and managing complications related to NCDs. An individual’s KAP of healthy lifestyle determines the development and management of NCDs. This is potentially the first comprehensive study describing the associations of KAP elements of healthy diet and physical activity in Qatar’s population. The study found that individuals demonstrated high level of knowledge (healthy diet mean score = 75.68, physical activity mean score = 66.6) and positive attitude (healthy diet mean score = 74.14, physical activity mean score = 76.9) of healthy diet and physical activity, however, they did not practice it (healthy diet mean score = 49.58, physical activity mean score = 9.56). The findings highlight that being informed and having the intention may not necessarily lead to practice of healthy lifestyle. This implies socioeconomic, cultural and personal factors play an important role in shaping people’s behaviours.

The study highlights potential barriers in translating knowledge into practice in primary care registered population in Qatar. There is substantial literature from other countries which report similar findings signifying the lack of transition from acquisition of knowledge of healthy lifestyles, developing positive attitudes towards the concept to eventually adopting real life healthy lifestyle practices which can determine the health outcomes.^13–18^

This study did not find any association between gender, BMI and educational status with the mean total score of healthy diet practice. On the contrary a study reporting the results of two prospective cohort studies reported strong associations with health lifestyle practices and health outcomes with individuals with low socio-economic status.^19^ Interestingly, this study found no statistical significance between the association of nationality and healthy lifestyle practices. Whereas evidence suggests that the dietary preferences can also vary between different geographical settings with some ethnicity’s preferences of consuming specific unhealthy diets.^20,21^ This may be attributed to various multi-faceted factors such as cultural preferences, cooking practices or individual work life circumstances such as lack of time which may lead to consumption of quick and easier option of eating fast food.^22,23^

The study findings suggest that attitude exerts a measurable, but modest influence on the adoption of healthy lifestyle practices. This highlights attitude as a proximal determinant of practice, while also indicating that its effect may be constrained by wider contextual and structural factors.^13^ This finding further signifies the constraining influence of structural and contextual factors such as time scarcity, environmental limitations, and competing social demands that hinder the translation of intention into action.^24–26^

Variation in lifestyle behaviors by marital status further highlights this complexity, with marriage associated with healthier dietary practices but lower physical activity levels. Moreover, the absence of consistently better practices among individuals reporting diabetes or dyslipidemia suggests that clinical diagnosis alone does not reliably prompt sustained lifestyle modification. Together, these findings substantiate the recommendations in existing literature which highlight the need for behaviorally informed, context-responsive primary care and public health strategies that move beyond information provision to effectively reduce NCD risk.^26,27^

Behaviour change is essential to prevent and manage NCDs. Several factors influence behaviour change which should be considered when developing and implementing public health interventions. The main factors associated with practicing healthy lifestyles identified by recent literature can be broadly categorized as socio-economic and environmental factors, acquired learning influencing individual behaviors and preferences towards diet, cultural influences, individual motivation levels towards practicing healthy lifestyles, access to health information and technology, associated co-morbidities and overall quality of life and lack of peer and family support.^19,23,28–34^ Studies also suggest that an individual’s practicing healthy lifestyles benefits from peer support groups and family support and have higher compliance as compared to individuals who practice in isolation without such support mechanism.

### Strengths and limitations

A key strength of the study is that the participants present a diverse cohort group which increases the generalizability of the findings of the strength. Another key strength of the study is that the KAP tool utilized in the study was culturally adapted and pilot tested based on key theoretical evidence-based steps. The methodology of tailoring the tool was peer reviewed and published. This strengthens the validity of the tool utilized in the study. The multistage random sampling approach and inclusion of participants from multiple geographic regions improve the generalisability of the results to the wider primary care population. Rigorous data collection procedures, including interviewer-administered questionnaires and standardised scoring, minimise information bias and ensure data quality. One of the limitations of the study is that the cross-sectional design precludes any inference of causality between knowledge, attitudes, and practices, or between these factors and health outcomes. Self-reported data may be subject to recall bias and social desirability bias, potentially leading to overestimation of positive behaviours. The study did not explore in-depth the barriers and facilitators to behaviour change, which could be addressed in future qualitative research.

## Implications for clinical practice

The findings of this study have significant implications for clinical practice in primary care settings in Qatar and similar contexts. Despite high levels of knowledge and positive attitudes towards healthy diet and physical activity among the primary care registered population, the translation of this knowledge and attitude into actual healthy lifestyle practices remains limited. This gap underscores the need for a paradigm shift in clinical practice from solely providing information to actively supporting behaviour change.

1. Behaviour Change Interventions: The results highlight that knowledge and positive attitudes alone are insufficient to drive healthy lifestyle behaviours. Primary care practitioners should incorporate evidence-based behaviour change techniques into routine consultations. This may include motivational interviewing, goal setting, personalised feedback, and regular follow-up to address barriers and reinforce progress.
2. Culturally Tailored Approaches: Given the diverse population in Qatar, interventions must be culturally and linguistically adapted. Clinicians should be aware of cultural preferences, dietary habits, and social norms that may influence patients’ ability to adopt healthy behaviours. Utilising culturally relevant educational materials and involving family members in counselling sessions may enhance effectiveness.
3. Interdisciplinary Collaboration: Addressing lifestyle-related risk factors for NCDs requires a multidisciplinary approach. Collaboration between physicians, dietitians, physiotherapists, and health educators can provide comprehensive support for patients. Integrating lifestyle counselling into routine care pathways and leveraging community resources can help bridge the gap between knowledge and practice.
4. System-Level Support: The study’s findings suggest the need for system-level changes, such as the implementation of structured lifestyle intervention programmes within primary care. Electronic health records can be used to prompt clinicians to assess and document lifestyle behaviours, set reminders for follow-up, and monitor patient progress over time.
5. Patient Empowerment and Engagement: Empowering patients to take an active role in their health is crucial. Shared decision-making, self-monitoring tools, and peer support groups can enhance patient engagement and accountability. Clinicians should foster a supportive environment that encourages patients to set realistic goals and celebrate incremental successes.

## Conclusion

This study provides evidence that having knowledge and attitude of healthy lifestyle does not necessarily result in its practice. Research suggests that some individuals may be disadvantaged from performing health behaviors due to socio-economic and environmental factors. Identification of these factors and a comprehensive analysis in primary care registered population in Qatar is necessary. These findings will provide critical and timely insights necessary to identify and inform primary care and public health interventions related to healthy lifestyle. In addition, the modest influence of attitudes and the limited behavioral impact of cardiometabolic diagnoses indicate that information and clinical awareness alone are insufficient to produce sustained lifestyle change. These findings emphasize the need for structured, behaviorally informed, and context-responsive interventions within primary care to effectively reduce non-communicable disease risk. Looking forward, primary care in Qatar and similar settings must evolve to address the complex interplay of knowledge, attitudes, and behaviors’ influencing NCD risk. Personalized Care, Continuous Professional Development, Research and Evaluation, and Policy and Advocacy perspectives are recommended.

## Supplemental material

Supplemental material - Knowledge, attitude and practice of healthy lifestyle among primary care registered population in Qatar: A cross sectional study Supplemental material for Knowledge, attitude and practice of healthy lifestyle among primary care registered population in Qatar: A cross sectional study by Mohamed Ahmed Syed, Ahmed Sameer Al Nuaimi, Abduljaleel Abdullatif Zainel, Hafiz Ahmed Mohamed and Muslim Abbas Syed in Journal of Public Health Research.

## Data Availability

The datasets used and/or analysed during the current study are available from the corresponding author on reasonable request.*

## References

[1] Shu J Jin W . Prioritizing non-communicable diseases in the post-pandemic era based on a comprehensive analysis of the GBD 2019 from 1990 to 2019. Scientific Reports 2023; 13: 13325. 10.1038/s41598-023-40595-7 37587173 PMC10432467

[2] Noncommunicable diseases. https://www.who.int/news-room/fact-sheets/detail/noncommunicable-diseases (2022, accessed 06 August 2023).

[3] Li Y Fan X Wei L , et al. The impact of high-risk lifestyle factors on all-cause mortality in the US non-communicable disease population. BMC Public Health 2023; 23: 422. 10.1186/s12889-023-15319-1 36864408 PMC9979572

[4] Hambleton IR Caixeta R Jeyaseelan SM , et al. The rising burden of non-communicable diseases in the Americas and the impact of population aging: a secondary analysis of available data. The Lancet Regional Health–Americas 2023; 21: 100483. 10.1016/j.lana.2023.100483 37065858 PMC10090658

[5] Omotayo O Maduka CP Muonde M , et al. The rise of non-communicable diseases: a global health review of challenges and prevention strategies. International Medical Science Research Journal 2024; 4: 74–88. 10.51594/imsrj.v4i1.738

[6] Tackling NCDs: best buys and other recommended interventions for the prevention and control of noncommunicable diseases, second edition. Report no. WHO/NMH/NVI/17.9. Geneva: World Health Organization, 2024. Licence: CC BY-NC-SA 3.0 IGO.

[7] Mohamed AS , Ahmed SA , Abdul Jaleel Z , et al. Prevalence of non-communicable diseases by age, gender and nationality in publicly funded primary care settings in Qatar. BMJ Nutrition, Prevention & Health 2019; 2: 20. 10.1136/bmjnph-2018-000014 PMC7678476 33235953

[8] Elmusharaf K Stanton R Chestnov R , et al. Prevention and Control of Non- Communicable Diseases in Qatar: The Case for Investment. Geneva: UNDP, WHO, UNIATF, GHC, 2021. https://www.undp.org/sites/g/files/zskgke326/files/2024-02/qatar_ncd_ic_eng.pdf

[9] Elmusharaf K Grafton D Jung JS , et al. The case for investing in the prevention and control of non-communicable diseases in the six countries of the Gulf Cooperation Council: an economic evaluation. BMJ Global Health 2022; 7: e008670. 10.1136/bmjgh-2022-008670 PMC9161070 35649631

[10] Noncommunicable diseases country profiles 2018. World Health Organization, 2018. Available from https://www.who.int/publications/i/ite, ​Licence: CC BY-NC-SA 3.0 IGO.

[11] Awad SF O'Flaherty M Critchley J , et al. Forecasting the burden of type 2 diabetes mellitus in Qatar to 2050: A novel modeling approach. Diabetes Res Clin Pract 2018; 137: 100–108, 2017/11/28. 10.1016/j.diabres.2017.11.015 29175341

[12] Alnuaimi AS Syed MA Zainel AA , et al. Cultural & region-specific adaptation of KAP (Knowledge, attitude, and practice) tool to capture healthy lifestyle within primary care settings. PLoS One 2024; 19: e0312852, 2024/12/19. 10.1371/journal.pone.0312852 39700224 PMC11658580

[13] Naaz S . Knowledge, attitude and practices pertaining to healthy lifestyle in prevention and control of chronic diseases: a rapid review. International Journal of Community Medicine and Public Health 2021; 8: 5106. 10.18203/2394-6040.ijcmph20213822

[14] Ma L Liu H Tao Z , et al. Knowledge, Beliefs/Attitudes, and practices of rural residents in the prevention and control of COVID-19: an online questionnaire survey. The American journal of tropical medicine and hygiene 2020; 103: 2357–2367. 10.4269/ajtmh.20-0314 33124537 PMC7695081

[15] Yenit MK Kolbe-Alexander TL Gelaye KA , et al. An Evaluation of Community Health Workers’ knowledge, attitude and personal lifestyle Behaviour in Non-communicable Disease Health Promotion and Their Association with self-efficacy and NCD-Risk perception. International Journal of Environmental Research and Public Health 2023; 20: 5642. 10.3390/ijerph20095642 37174162 PMC10178727

[16] Van Zyl S Kruger WH Walsh CM . Chronic diseases of lifestyle curriculum: Students’ perceptions in primary health care settings. African Journal of Primary Health Care & Family Medicine 2023; 15: 3775. 10.4102/phcfm.v15i1.3775 36744458 PMC9900301

[17] Alhowaymel FM Abdelmalik MA Mohammed AM , et al. Knowledge, attitudes, and practices of hypertensive patients towards stroke prevention among rural population in Saudi Arabia: A cross-sectional study. SAGE open nursing 2023; 9: 23779608221150717. 10.1177/23779608221150717 36643783 PMC9834414

[18] Vainauskienė V Vaitkienė R . Enablers of patient knowledge empowerment for self-management of chronic disease: an integrative review. International journal of environmental research and public health 2021; 18: 2247. 10.3390/ijerph18052247 33668329 PMC7956493

[19] Zhang Y-B Chen C Pan X-F , et al. Associations of healthy lifestyle and socioeconomic status with mortality and incident cardiovascular disease: two prospective cohort studies. Bmj 2021; 373: n604. 10.1136/bmj.n604 33853828 PMC8044922

[20] Anand S Bradshaw C Prabhakaran D . Prevention and management of CVD in LMICs: why do ethnicity, culture, and context matter? BMC medicine 2020; 18: 1–5. 10.1186/s12916-019-1480-9 31973762 PMC6979081

[21] Gherasim A Arhire LI Niță O , et al. The relationship between lifestyle components and dietary patterns. Proceedings of the Nutrition Society 2020; 79: 311–323. 10.1017/S0029665120006898 32234085 PMC7663317

[22] Yau A Adams J White M , et al. Differences in diet quality and socioeconomic patterning of diet quality across ethnic groups: cross-sectional data from the HELIUS Dietary Patterns study. European journal of clinical nutrition 2020; 74: 387–396. 10.1038/s41430-019-0463-4 31292529 PMC7062636

[23] Tao M-H Liu J-L Nguyen U-SD . Trends in diet quality by race/ethnicity among adults in the United States for 2011–2018. Nutrients 2022; 14: 4178. 10.3390/nu14194178 36235830 PMC9570938

[24] Aditi M Raychaudhuri D John D . A Study on the Importance of Dietary Habits, Nutritional Patterns, and Lifestyle Behaviours as Predictors of Chronic Disease Symptoms: A Cross-Sectional KAP Study Across Different Age Groups in Delhi NCR, India. South Asian Research Journal of Nursing and Healthcare 2026; 8: 15–25. 10.36346/sarjnhc.2026.v08i01.004

[25] Fadhil A Gabrielli S . Addressing challenges in promoting healthy lifestyles: the al-chatbot approach. Proceedings of the 11th EAI International Conference on Pervasive Computing Technologies for Healthcare. Association for Computing Machinery, 2017, pp. 261–265.

[26] Glasgow RE Goldstein MG Ockene JK , et al. Translating what we have learned into practice. Principles and hypotheses for interventions addressing multiple behaviors in primary care. Am J Prev Med 2004; 27: 88–101. 10.1016/j.amepre.2004.04.019 15275677

[27] Syed MA Syed MA Lee AC . Integrated care for chronic diseases: An evolutionary step for emerging primary health care systems. Public Health 2024; 237: 456–457. 20241116. 10.1016/j.puhe.2024.11.007 39549560

[28] Mamurov B Mamanazarov A Abdullaev K , et al. Acmeological approach to the formation of healthy lifestyle among university students. In: III international scientific congress society of ambient intelligence 2020 (ISC-SAI 2020). Atlantis Press, 2020, pp. 347–353.

[29] Tandon A Dhir A Kaur P , et al. Why do people buy organic food? The moderating role of environmental concerns and trust. Journal of Retailing and Consumer Services 2020; 57: 102247. 10.1016/j.jretconser.2020.102247

[30] Budreviciute A Damiati S Sabir DK , et al. Management and prevention strategies for non-communicable diseases (NCDs) and their risk factors. Frontiers in public health 2020; 8: 574111. 10.3389/fpubh.2020.574111 33324597 PMC7726193

[31] Biswas SS . Role of chat gpt in public health. Annals of biomedical engineering 2023; 51: 868–869. 10.1007/s10439-023-03172-7 36920578

[32] Jabbar M Yusoff MM Shafie A . Assessing the role of urban green spaces for human well-being: A systematic review. GeoJournal 2022; 87: 1–19. 10.1007/s10708-021-10474-7 34305268 PMC8290137

[33] Nguyen P-Y Astell-Burt T Rahimi-Ardabili H , et al. Green space quality and health: a systematic review. International journal of environmental research and public health 2021; 18: 11028. 10.3390/ijerph182111028 34769549 PMC8582763

[34] Gassner L Zechmeister-Koss I Reinsperger I . National strategies for preventing and managing non-communicable diseases in selected countries. Frontiers in Public Health 2022; 10: 838051. 10.3389/fpubh.2022.838051 35223747 PMC8867176

