# Supplementary material for: Knowledge, attitude and practice of healthy lifestyle among primary care registered population in Qatar: A cross sectional study
Source: J Public Health Res. 2026 Jun 5;15(2):22799036261457516. doi: 10.1177/22799036261457516 (PMC13241617; doi:10.1177/22799036261457516)
Supplement: Supplemental material - Knowledge, attitude and practice of healthy lifestyle among primary care registered population in Qatar: A cross sectional study [file sj-pdf-1-phj-10.1177_22799036261457516.pdf]

Table S1: Missing Data Across Selected Study Variables

	N	%
**Outcome variables**		
Total Knowledge score-Healthy diet (max=100)	50	5.3
Total Attitude score-Healthy diet (max=100)	2	0.2
Total Practice score-Healthy diet (max=100)	198	21.1
Total Knowledge score-Physical activity (max=100)	41	4.4
Total Attitude score-Physical activity (max=100)	50	5.3
Total score of physical activity practice (max=100)	27	2.9
**Explanatory variables**		
Age group (years)	43	4.6
Obesity (BMI >=30 kg/m2)	34	3.6
Gender	25	2.7
Ever married	17	1.8
Educational level	11	1.2

Table S2: Proportion Reporting Correct Knowledge About Healthy Diet

	N	%
Correctly identified as healthy dietary habit (Total N=934)		
Balanced variety of the following food categories: Vegetables, Fruit, Legumes, Cereals & Starchy Vegetables like potatoes, Milk, Dairy Products, and Fish, Poultry, Meat	890	95.3
Eating more vegetables	827	88.5
Lots of fresh fruit	813	87
Using less fat in cooking	807	86.4
Less sugar/sweet foods	786	84.2
Eating main meals (Breakfast, lunch and/or dinner) regularly and not snacking in between	772	82.7
Lowering salt and salty sauces consumption	755	80.8
Keep regular hours for meals	751	80.4
Choose skinless poultry and lean cuts of meat	690	73.9
Avoid canned/processed food (Mortadella, Salami, Sausages, Chips)	677	72.5
Substitute white bread with whole grain (brown) bread, in addition to eating cereals	621	66.5
If you cannot avoid snacks then choose unsalted nuts and seeds as part of a healthy snack	573	61.3
Read food labels to choose nutritious foods with high fiber content and to avoid bad fat (hydrogenated or trans-fat).	548	58.7
Read food labels to avoid food with high calory/ energy content.	513	54.9
Its preferrable to choose sources other than dairies as a source of calcium and vitamin D rich foods like almonds and chickpeas	512	54.8
Avoid carbohydrate rich food (Wheat, Starchy vegetables like potatoes)	478	51.2
Correctly identified as healthy food items (Total N=935)		
Fresh Vegetables, Vegetable sautés, soup and curries	880	94.1
Fresh whole fruits	876	93.7
Meat/Chicken/Fish	859	91.9
Salad	858	91.8
Whole grain (bread) bread	787	84.2
Rice	751	80.3
Correctly identified as unhealthy food items (Total N=935)		
Sweetened soft drinks (Fanta, Cola, Pepsi …etc)	874	93.5
Chips	862	92.2
Mortadella, Salami, Sausage, , pizza	840	89.8
Jam, Desserts	807	86.3
Doughnuts, cookies	804	86
Beef/chicken burgers	724	77.4
Traditional sweets (Baklava)	724	77.4
Deep fried vegetables	670	71.7
White bread/ pasta	469	50.2
Any freshly prepared food which is available	198	21.2
Correctly identified health risk associated with obesity and unhealthy food (Total N=905)		

	N	%
Hypertension	817	90.3
cardiovascular diseases	814	89.9
type 2 diabetes	811	89.6
Bad teeth	739	81.7
Osteoporosis	494	54.6
Degenerative joint problems like osteoarthritis	482	53.3
Correctly defined Junk Food as (Total N=916)		
Food that is high in calories from sugar and/or fat, and possibly also sodium (table salt for example)	717	78.3
Little dietary fiber, protein, vitamins, minerals or other important forms of nutritional value	612	66.8
Correctly identified as choices for healthy lifestyle (Total N=915)		
Eat home-made food more often and explore healthy ways to prepare traditional foods	851	93
You should always watch your waist circumference in addition to height and weight.	714	78
Consuming sugar sweetened drinks is an important cause of weight gain	669	73.1
Maintain a healthy weight. You should aim to a body mass index between 18.5 to 25 kg/m2	595	65
Correctly identified the following as high salt content food items (Total N=927)		
Pickled vegetables	663	71.5
Chips	635	68.5
Tabasco and Soy sauce	564	60.8
Salty laban (Ayran)	527	56.9
Mortadella, Salami, Sausage	505	54.5

Table S3: Proportion Reporting Favourable Attitude towards a Healthy Diet

	N	%
**Disagree/strongly disagree with a negatively phrased attitude item (Total N=938)**		
Overweight people are healthier	831	88.6
Overweight people are more attractive	806	85.9
Healthy food is for sick people	760	81.0
If you don't have any health problems, you can eat whatever you like	661	70.5
Soft drinks (other than fresh juice) are good in the summer	656	69.9
If you do enough exercise, you can eat whatever you like	627	66.8
Healthy food is not tasty	456	48.6
**Agree/strongly agree with a positively phrased attitude item (Total N=938)**		
Healthy food is important	924	98.5
I want to drink more pure water during the day	844	90.0
I want to improve my food intake pattern	827	88.2
I want to improve my family's food pattern?	797	85.0
lowering the salt in diet is important for health	789	84.1
Healthy foods are enjoyable	688	73.3
I really care about what I eat	670	71.4
I wouldn't let my children eat junk food	436	46.5
I always think of the calories in what I eat	355	37.8

Table S4: Proportion Reporting Recommended Healthy Diet Practice

Healthy diet recommended practice (Total N=886)	N	%
Monitoring body weight during the last 6 months	734	82.8
Buy ingredients and cook at home	693	78.2
Never/rarely eating processed food high in salt	578	65.2
Never/rarely adding salty sauce to food before/during eating	549	62
Drinking natural fruit or vegetable juice less than once a week	288	32.5
Recommended vegetable consumption	73	8.2
Recommended fruit consumption	41	4.6

Table S5: Multiple linear regression model for the total healthy diet practice score (max=100) as the dependent (outcome) variable and selected explanatory variables.

	Unstandardized Regression Coefficients	95% confidence interval	Standardized Coefficients	P
(Constant)	28.89	(18.77 to 39.01)		<0.001
Associated comorbidities				
Asthma	-2.10	(-6.69 to 2.49)	-0.04	0.37[NS]
Cardiovascular Diseases (heart attack, angina, stroke)	0.22	(-8.55 to 8.99)	0.002	0.96[NS]
Raised blood pressure (hypertension)	0.54	(-3.54 to 4.62)	0.01	0.8[NS]
Diabetes	4.20	(-0.38 to 8.79)	0.08	0.07[NS]
Raised blood Cholesterol (dyslipidemia)	0.46	(-4.04 to 4.95)	0.01	0.84[NS]
Healthy diet Total Knowledge score (max=100)	0.003	(-0.12 to 0.12)	0.002	0.96[NS]
Healthy diet Total Attitude score (max=100)	0.23	(0.14 to 0.31)	0.22	<0.001
Male gender compared to female	-0.07	(-2.63 to 2.49)	0.002	0.96[NS]
Being obese (BMI >=30 kg/m2)	-1.98	(-4.99 to 1.03)	-0.05	0.2[NS]
Ever married compared to single	3.81	(0.23 to 7.39)	0.10	0.037
Age group (years)				
(30-39) compared to <30	3.54	(-0.39 to 7.46)	0.11	0.08[NS]
(40-49) compared to <30	4.68	(0.34 to 9.02)	0.12	0.035
50+ compared to <30	4.22	(-1.06 to 9.51)	0.09	0.12[NS]
Nationality groups				
Qatari nationality compared to Other (miscellaneous) nationality	-1.07	(-5.01 to 2.87)	-0.03	0.59[NS]
South-eastern Asia compared to Other (miscellaneous) nationality	-3.25	(-7.7 to 1.2)	-0.07	0.15[NS]
Southern Asia compared to Other (miscellaneous) nationality	2.38	(-1.13 to 5.88)	0.07	0.18[NS]
Educational attainment				
Completed 12 classes/Trade/technical/vocational qualification compared to Less educated (did not complete the secondary school)	-2.59	(-6.85 to 1.67)	-0.07	0.23[NS]
University Diploma/bachelor's degree/ Postgraduate degree compared to Less educated (did not complete the secondary school)	-3.49	(-7.55 to 0.57)	-0.10	0.09[NS]

R Square=0.128

P (Model)<0.001

TableS6: Proportion Reporting Correct Knowledge About Physical Activity

	N	%
**Correctly identified the recommended amount of physical activity (Total N=556)**		
Adults willing to lead a healthy life should engage in regular moderate-intensity physical activities (like walking and cycling) for a minimum of 30 min a day for 5 days per week	461	82.9
Adults willing to lead a healthy life should engage in regular vigorous-intensity aerobic physical activity (like running and swimming) for a minimum of 20 min a day for 3 days per week	208	37.4
**Correctly identified the following known benefits of regular physical activities (Total N=908)**		
lower blood sugar in people with diabetes	836	92.1
Relieve stress	809	89.1
Physical activity is as important as healthy food in reducing the risk of over 25 chronic health conditions	705	77.6
Increase the "good" cholesterol levels	676	74.4
Older people can protect their bones from osteoporosis by walking more frequent.	592	65.2

Table S7: Proportion Reporting Favourable Attitude towards Physical Activity.

Disagree/strongly disagree with a negatively phrased attitude item

1. I don't want to do more physical activity because it increases my appetite and increases my weight
2. I don't need to practice more physical exercise because I don't feel the need to loose weight
3. When I eat less I don't need to be physically active

Agree/strongly agree with a positively phrased attitude item

4. Physical activity is important for maintaining a good health
5. Low physical activity is linked to overweight and obesity
6. I enjoy physical activity
7. TV, electronic games, smart phone and computer use should be restricted as much as possible

Table S8: Proportion Reporting Recommended Physical Activity

	N	%
Achieving the recommended level of practicing physical exercise (Total N=940)		
Physical exercise of any intensity	475	50.5
Moderate intensity physical activity	290	30.9
Low-intensity physical activity	225	23.9
Vigorous physical activity	173	18.4

Table S9: Multiple linear regression model for the total physical activity practice score (max=100) as the dependent (outcome) variable and selected explanatory variables.

	Unstandardized Regression Coefficients	95% confidence interval	Standardized Coefficients	P
(Constant)	-1.09	(-6.79 to 4.62)		0.71[NS]
Associated comorbidities				
Asthma	2.06	(-1.11 to 5.24)	0.05	0.2[NS]
Cardiovascular Diseases (heart attack, angina, stroke)	-2.75	(-8.56 to 3.06)	-0.04	0.35[NS]
Raised blood pressure (hypertension)	2.02	(-0.93 to 4.96)	0.06	0.18[NS]
Diabetes	-0.85	(-4.2 to 2.5)	-0.02	0.62[NS]
Raised blood Cholesterol (dyslipidemia)	1.74	(-1.49 to 4.96)	0.04	0.29[NS]
Physical activity Total Knowledge score	0.04	(-0.01 to 0.09)	0.06	0.12[NS]
Physical activity Total Attitude score	0.13	(0.07 to 0.18)	0.17	<0.001
Male gender compared to female	-0.75	(-2.67 to 1.17)	-0.03	0.44[NS]
Being obese (BMI >=30 kg/m2)	-1.47	(-3.68 to 0.74)	-0.05	0.19[NS]
Ever married compared to single	-4.35	(-7.06 to -1.64)	-0.14	0.002
Age group (years)				
(30-39) compared to <30	-0.18	(-3.12 to 2.75)	-0.01	0.9[NS]
(40-49) compared to <30	1.11	(-2.15 to 4.37)	0.04	0.5[NS]
50+ compared to <30	-0.71	(-4.72 to 3.3)	-0.02	0.73[NS]
Nationality groups				
Qatari nationality compared to Other (miscellaneous) nationality	-3.29	(-6.23 to -0.35)	-0.11	0.028
South-eastern Asia compared to Other (miscellaneous) nationality	-3.00	(-6.35 to 0.35)	-0.08	0.08[NS]
Southern Asia compared to Other (miscellaneous) nationality	-0.29	(-2.88 to 2.31)	-0.01	0.83[NS]
Educational attainment				
Completed 12 classes/Trade/technical/vocational qualification compared to Less educated (did not complete the secondary school)	2.03	(-1.12 to 5.18)	0.07	0.21[NS]
University Diploma/bachelor's degree/ Postgraduate degree compared to Less educated (did not complete the secondary school)	3.92	(0.94 to 6.9)	0.15	0.01

R Square=0.098

P (Model)<0.001
